# Metformin and the gastrointestinal tract

**DOI:** 10.1007/s00125-015-3844-9

**Published:** 2016-01-16

**Authors:** Laura J. McCreight, Clifford J. Bailey, Ewan R. Pearson

**Affiliations:** Pearson Group, Division of Cardiovascular and Diabetes Medicine, School of Medicine, University of Dundee, Ninewells Hospital, Mailbox 12, Level 5, Dundee, DD1 9SY UK; School of Life and Health Sciences, Aston University, Birmingham, UK

**Keywords:** Bile acids, DPP-4, GLP-1, Gut/intestine, Lactate, Metformin, Microbiome, OCT1, Review, Serotonin, Uptake

## Abstract

Metformin is an effective agent with a good safety profile that is widely used as a first-line treatment for type 2 diabetes, yet its mechanisms of action and variability in terms of efficacy and side effects remain poorly understood. Although the liver is recognised as a major site of metformin pharmacodynamics, recent evidence also implicates the gut as an important site of action. Metformin has a number of actions within the gut. It increases intestinal glucose uptake and lactate production, increases GLP-1 concentrations and the bile acid pool within the intestine, and alters the microbiome. A novel delayed-release preparation of metformin has recently been shown to improve glycaemic control to a similar extent to immediate-release metformin, but with less systemic exposure. We believe that metformin response and tolerance is intrinsically linked with the gut. This review examines the passage of metformin through the gut, and how this can affect the efficacy of metformin treatment in the individual, and contribute to the side effects associated with metformin intolerance.

## Introduction

Metformin—dimethylbiguanide—is an oral glucose-lowering agent. Its origins can be traced to *Galega officinalis,* also known as French lilac or goat’s rue [1]. In the early 20th century it was noted to lower blood glucose concentrations in animals, but it was not until the 1950s that Jean Sterne studied dimethylbiguanide and subsequently developed ‘Glucophage’ [2].

Over the last 15 years, metformin has become the first-line agent for the treatment of type 2 diabetes, as noted in several international guidelines, including the ADA-EASD guidelines [3]. Metformin has had a chequered history—it was initially eclipsed by phenformin, which was withdrawn in the late 1970s after it was discovered to be associated with lactic acidosis [4]. The lower propensity of metformin for hyperlactataemia [5] and success in several large randomised controlled clinical trials, such as the UK Prospective Diabetes Study (UKPDS) [6], confirmed its clinical benefit. It is widely recognised that metformin improves glycaemic control with a good safety profile, weight neutrality or weight loss, lack of associated hypoglycaemia, reduced cardiovascular mortality and low cost [3]. However, a large proportion of patients cannot tolerate the medication in adequate amounts because of its associated side effects. Up to 25% of patients suffer metformin-associated gastrointestinal (GI) side-effects, with approximately 5% unable to tolerate metformin at all [7]. In addition to this interindividual variation in side effects, there is variability in the efficacy of metformin. There are likely to be a number of factors to account for this variability in efficacy, for example, our group (Zhou et al) recently established that the glycaemic response to metformin is moderately heritable, i.e. due in part to genetic variation [8].

In this review we will focus on the effects of metformin on the gut and how its action within the intestine and on the intestinal enterocytes can explain at least some of the glucose-lowering effects of metformin, the increase in lactate concentrations and GI side-effects of this commonly used drug.

**Table:** 

**Summary of key points**
• Metformin uptake is saturable and dose-dependent, consistent with a predominantly transporter-dependent mechanism. Uptake and tolerance could be affected by genetic variation in the transporters, or by transporter-inhibiting drugs
• Metformin increases glucose uptake in the intestine, and subsequently increases lactate concentrations within the enterocyte. This may contribute to metformin intolerance
• Metformin increases plasma GLP-1 concentrations, though the mechanism is unclear. This could be direct or indirect. The effect of metformin on DPP-4 is likely to be small
• Metformin may, in part, utilise a gut–brain–liver axis to exert its pharmacodynamic effect
• Metformin increases the bile acid pool within the intestine, which may affect stool consistency, GLP-1 secretion, cholesterol levels and the microbiome
• Metformin alters the microbiome, which may improve glucose tolerance, but, conversely, may play a role in metformin intolerance

## Intestinal transport of metformin

Metformin is usually taken orally as the hydrochloride salt, in a tablet formulation. It exists largely as a hydrophilic cationic species at physiological pH, and has low lipid solubility, making rapid passive diffusion of metformin through cell membranes unlikely [4, 9]. Absorption of immediate-release formulations of metformin is largely confined to the small intestine, with negligible absorption in the stomach or large intestine [4, 10, 11]. In humans, intravenous administration of metformin results in rapid renal elimination, with little or no metformin detectable in the faeces [10], consistent with negligible biliary or GI secretion of metformin. However, in a mouse model of diabetes, intravenous administration of metformin does lead to accumulation of metformin in the enterocytes—most notably in the small intestine [12]. Oral bioavailability of metformin is between 50% and 60%, with approximately 30% dose recovery of unchanged metformin from faeces. Bioavailability is affected by gastric motility and may be reduced by high-fat meals [4]. The metformin concentration in the jejunum peaks at 500 μg/g, 30–300 times greater than plasma concentrations [13], highlighting the small intestine as an important site of metformin uptake.

Modified-release formulations of metformin have been developed to spread the absorption of metformin along the gut and thereby reduce local concentrations of the drug, with the aim of increasing its tolerability. Metformin MR (modified-release) uses a dual polymer matrix to delay the transit and slow the release of metformin in the gut. On contact with fluid from the GI tract, the tablet swells, and the metformin is released as the polymer gradually breaks down [14]. A new metformin formulation has recently been developed, metformin DR (delayed-release), which is formulated to target the ileum via pH-dependent dissolution of the tablet. Compared with metformin IR (immediate-release) or metformin XR (extended-release), the bioavailability of metformin DR is lower, yet its glucose-lowering efficacy is similar, despite lower systemic metformin exposure [15]. This again highlights the ileum as a site of uptake and as an important site of action of metformin in lowering blood glucose.

Metformin uptake is saturable and dose-dependent [10, 16], consistent with the theory that it is mostly transporter-dependent. Studies in Caco-2 cell monolayers (a cellular model of human intestinal epithelium) have shown that metformin is efficiently taken up across the apical (luminal-facing) surface of enterocytes via bidirectional transporters, but that efflux across the basolateral surface of enterocytes is limited, resulting in the accumulation of metformin in the epithelium [16], possibly accounting for the greatly increased metformin concentration seen in these cells. To account for the presence of metformin in the portal circulation, some paracellular uptake was postulated, with metformin diffusing passively.

Several transporters, for which metformin is a likely substrate, have been identified e.g. organic cation transporter (OCT) 1–3, plasma membrane monoamine transporter (PMAT), multidrug and toxin extrusion protein (MATE) 1–2, serotonin transporter (SERT) and high-affinity choline transporter (CHT). OCTs are members of solute carrier family 22 (SLC22), initially described in 1994, and encoded on chromosome 6q26 [17]. OCTs are expressed in several tissues, including the intestine, liver, kidney, brain, muscle and heart. OCT1 is predominantly expressed in the liver, but plays an important role in the transfer of cations, including metformin, from the gut lumen to the interstitium [18]. Although initial reports localised OCT1 to the basolateral membrane [19], more recent reports place OCT1 on the apical surface of intestinal epithelial cells [20, 21]. OCT2 is expressed mainly in the kidney and is partly responsible for the renal excretion of metformin [18]. OCT3 is mainly expressed in the skeletal muscle, but is also expressed in the intestine. Interestingly, OCT3 is associated with metformin uptake and efflux in the salivary glands, which may account for the dysgeusia associated with metformin treatment [22]. PMAT was originally identified as a monoamine transporter from the equilibrative nucleoside transporter (ENT) family, found predominantly in the brain and central nervous system [23, 24]. It was later recognised that PMAT was polyspecific and found in many tissues throughout the body, including the intestine, where it transports metformin with comparable affinity to the OCTs [25]. PMAT is localised to the tips of the mucosal epithelial layer, suggesting that PMAT has an integral role in metformin uptake [25].

To investigate which transporters are involved in the transport of metformin across the apical surface of enterocytes, Han et al used pharmacological inhibitors and knockdown studies in single transporter-expressing CHO (Chinese hamster ovary) cells and Caco-2 cell monolayers [20]. They concluded that the main transporters of metformin are OCT1, PMAT, SERT and CHT, accounting for approximately 25%, 20%, 20% and 15% of apical metformin transport, respectively. However, comparison of the expression of these transporters in Caco-2 cells vs human jejunum using western blot analysis revealed that the expression of all four proteins was significantly higher in Caco-2 cells, with the expression of CHT in human jejunum barely detectable [20]. Therefore, direct evidence of the in vivo contribution of CHT to metformin uptake is still lacking, leaving only OCT1, PMAT and SERT as likely metformin transporters in the human intestine. There are well-documented, relatively common loss-of-function variants in human OCT1, and genetic variation in OCT1 has been investigated by a number of groups in relation to pharmacokinetics, efficacy and GI intolerance [7, 26–29]. The impact of variants in PMAT [30] and SERT on these outcomes needs to be assessed.

### Intestinal OCT1 transport and metformin pharmacokinetics and efficacy

As OCT1 is probably apically expressed it is most likely to influence local concentrations of metformin in the gut (lumen and enterocytes), rather than transfer into the blood. Consistent with this interpretation, a recent study concluded that OCT1 variants with loss of function do not alter metformin concentrations in healthy volunteers titrated to 1 g/day of metformin [27]. An initial report that OCT1 variants altered the glycaemic response to metformin in a clinical trial [28] was not confirmed in a recent re-analysis that took account of the baseline HbA_1c_ level [29]. This is consistent with a report by our group (Zhou et al) on the Genetics of Diabetes Audit and Research Tayside Study (GoDARTS) data, where we found no effect on the glycaemic response to metformin in a large population of patients with type 2 diabetes [31].

### Intestinal OCT1 transport and GI intolerance with metformin

Given the role of OCT1 in potentially altering local concentrations of metformin, our group (Dujic et al) proposed that a reduction in function of OCT1 might alter the risk of GI intolerance in patients treated with metformin [7]. In this study, GoDARTS data were analysed to assess the effect of *OCT1* (also known as *SLC22A1*) genotype and the use of OCT1-inhibiting drugs on the incidence of metformin intolerance in a large population of patients with type 2 diabetes. Those with two reduced function *OCT1* alleles had a more than twofold increase in the odds for metformin intolerance (*p* < 0.001). This increased to an over fourfold increase in the odds for intolerance in patients with two reduced function OCT1 alleles who were also treated with OCT1-inhibiting drugs (*p* < 0.001) [7]. If confirmed by a clinical trial, this could inform the clinical application of metformin such that, for example, drugs known to interact with OCT1 could be replaced with alternatives, allowing improved tolerance to metformin and enabling optimal metformin dosing.

## Metformin, glucose uptake and anaerobic metabolism

### Metformin increases gut glucose uptake and utilisation

**Fig. 1:**
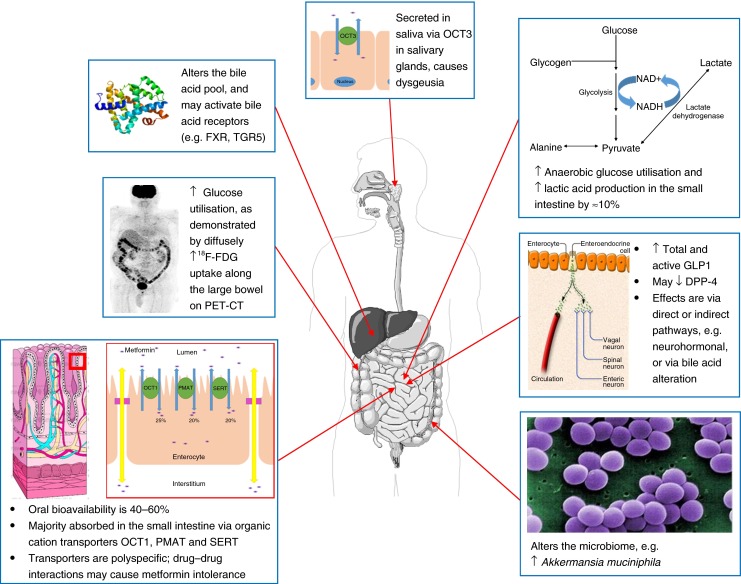
Some of the actions of metformin within the GI tract. The upward arrows indicate increases. DPP4, dipeptidyl peptidase-4; FXR, farnesoid X receptor; FDG, fluorodeoxyglucose; GLP-1, glucagon-like peptide-1; OCT, organic cation transporter; PMAT, plasma membrane monoamine transporter; SERT, serotonin transporter

It has been known for some time that metformin increases glucose uptake and utilisation in the human intestine, resulting in an increase in lactate production in enterocytes [12, 13]. Interestingly, the effect of metformin on gut glucose utilisation is well known to radiologists, as metformin use will interfere with imaging by positron emission tomography–computed tomography (PET-CT). This functional imaging technique uses positron-emitting ^18^F-fluorodeoxyglucose (^18^F-FDG) as a non-metabolised glucose analogue. The uptake of ^18^F-FDG is a marker of glucose uptake and, therefore, glucose utilisation, which is a measure of the metabolic activity of tissue. PET-CT is commonly used for the diagnosis, staging and monitoring of the response to treatment, of certain cancers [32]. However, patients taking metformin have diffusely increased ^18^F-FDG uptake in the colon and small intestine on PET-CT [33–35], as can be seen in Fig. 1. This confirms that metformin causes increased glucose uptake in the gut. It also poses a significant risk of false-negative imaging results when assessing for colonic or genitourinary tumours. For this reason it is recommended that metformin be discontinued for at least 48 h prior to a PET-CT procedure/scan [33, 35].

The mechanism whereby metformin increases gut glucose uptake and utilisation remains unclear [36–40]. In one study, metformin reduced the activity of sodium–glucose transporter 1 (SGLT1), but increased the recruitment of GLUT2 to the apical membrane of rat jejunum [38]; a study assessing the response to 5′-AMP-activated protein kinase (AMPK) activators (5-aminoimidazole-4-carboxamide ribonucleotide [AICAR] and metformin) suggests GLUT2 localisation to the apical membrane is mediated via rapid AMPK phosphorylation [38, 39]. The mechanism of glucose absorption and the role of GLUT2 in this process remains the subject of debate [40]. However, in fasting lean rodents GLUT2 is located on the basolateral membrane and is only recruited to the apical membrane of the enterocyte following postprandial glucose stimulation of SGLT1 [41], whereas insulin results in the internalisation of GLUT2. Interestingly, fasting obese humans, unlike fasting lean individuals, have persistent GLUT2 abundance in the apical membrane, signifying dysregulation of glucose sensing and transfer [41].

### Metformin and lactate production

As metformin treatment increases the uptake and utilisation of glucose, there is a subsequent increase in plasma lactate. Both the gut and liver are implicated as the main sources of metformin-related lactate production. In the gut, metformin increases the uptake and anaerobic metabolism of glucose, contributing to the rise in lactate associated with metformin treatment [12, 13, 42–49]. In rat hepatocytes, metformin inhibits mitochondrial glycerophosphate dehydrogenase, reducing the conversion of cytosolic lactate into pyruvate [42]. This build-up of intracellular lactate results in its release into the plasma. Observational data indicate that metformin causes a rise in fasting and average lactate levels [43], with an average increase (adjusted for plasma glucose and BMI) of 0.16 mmol/l compared with diet or sulfonylurea treatment.

Several studies have attempted to localise the lactate production associated with metformin treatment [44–49]. Perhaps most convincingly, rat studies in which plasma glucose and lactate levels were measured in the inferior vena cava, hepatic vein, hepatic portal vein and the aorta demonstrated that the plasma lactate concentration peaks in the hepatic portal vein, with a corresponding drop in the plasma glucose concentration. This implicates the intestine as the main site of metformin-associated anaerobic glucose utilisation and lactate production [44]. Ring biopsies taken from the jejunum and ileum of these rats after intrajejunal metformin infusion confirmed a 10% increase in intestinal lactate concentration [44]. These findings were confirmed in humans by assessment of lactate concentrations in jejunal biopsies incubated with and without metformin [13]. Cells incubated in solution with metformin had a higher lactate concentration (an increase of up to 35%) than those incubated in solution lacking metformin. These results provide corroborative evidence that metformin increases gut utilisation of glucose and subsequent lactate production. As a complementary explanation, lactate production with metformin treatment may be due to its action on the gut microbiome (see later), by inhibition of glycerophosphate dehydrogenase, which is found in some colonic bacteria. This raises the question of local lactate concentrations contributing to the GI symptoms associated with metformin intolerance. As yet there is no clinical evidence to support this theory, but it warrants further investigation.

## Gut-related peptides

### Metformin and glucagon-like peptide 1

Metformin treatment has been shown to increase the glucagon-like peptide 1 (GLP-1) concentration in both mouse and human studies [50–63]. GLP-1 is secreted from L cells, which are distributed throughout the intestine, but are highly concentrated in the ileum. GLP-1 is quickly degraded by dipeptidyl peptidase-4 (DPP4) in the intestinal mucosa and portal system. Metformin could potentially increase the GLP-1 concentration by increasing its secretion from L cells, and/or by reducing its breakdown by DPP4.

Several mouse and human in vivo studies have reported a metformin-associated reduction in DPP4 activity [50–57]. However, in vitro studies have yet to find a direct effect of metformin on the activity of DPP4 [58], suggesting that any effect of metformin on DPP4 activity may be indirect, possibly by influencing hepatic production of DPP4 [56, 64]. The results of a number of studies have suggested that any impact of metformin on DPP4 activity is likely to be small. First, metformin *increases* active GLP-1 levels in DPP4-deficient rats [59]. Second, DPP4 degrades both GLP-1 and gastric inhibitory polypeptide (GIP), yet metformin treatment in humans only increases the GLP-1 concentration, whereas DPP4 inhibitors increase both GLP-1 and GIP concentrations [60, 61]. Third, there is an additive effect on the GLP-1 concentration when metformin is added to the DPP4 inhibitor sitagliptin, suggesting a different mechanism of action of the two drugs [61].

Given that metformin is likely to have only a small impact on DPP4 activity, it is likely that the main impact of metformin on GLP-1 is to increase GLP-1 secretion. A study in mice reported that metformin increases the expression of precursor proteins such as preproglucagon and proglucagon in the large intestine, potentially increasing GLP-1 production and secretion [61]. Whilst one study on L cell-like lines did not support a direct effect of metformin on L cells to mediate this effect [60], a more recent study has reported a direct effect of metformin on these cells, where an increase in the expression of the gene encoding proglucagon by metformin is mediated via a β-catenin–TCF7L2-mediated mechanism [63, 65].

Metformin could also act *indirectly* to stimulate GLP-1 secretion, via alterations in the bile acid pool. Metformin inhibits the farnesoid X receptor (FXR) via an AMPK-mediated mechanism, resulting in reduced sensing and ileal absorption of bile acids [66]. The increase in the bile acid pool may then stimulate TGR5 bile acid receptors on the L cell [62, 67], causing an increase in GLP-1 secretion via mitochondrial oxidative phosphorylation and calcium influx [67].

### Metformin, serotonin and histamine

Metformin has some structural similarities with selective agonists of the 5-HT_3_ receptor and, as outlined earlier, is in part transported by SERT. Serotonin (5-HT) release from the intestine is associated with nausea, vomiting and diarrhoea—symptoms similar to those associated with metformin intolerance. Therefore, one possible mechanism for the GI intolerance associated with metformin may relate to altered transport of serotonin or to a direct serotonergic-like effect of metformin. Metformin stimulates the release of 5-HT from enterochromaffin cells collected by duodenal biopsy from metformin-naive individuals [68]; however, this effect is not mediated via the 5-HT_3_ receptor, as inhibition of the receptor does not alter the response. As an alternative explanation, it could be that metformin uptake via SERT or OCT1 results in reduced serotonin transport and resultant GI side-effects [69]. Finally, a recent paper has identified a potential role for metformin in inhibiting diamine oxidase, which is highly expressed in enterocytes and responsible for metabolism of histamine [69]. Histamine, like serotonin, is also associated with increased gut motility. More studies are required to establish the likely mechanism for GI intolerance to metformin and, importantly, how this key side effect can be avoided or treated.

## Metformin and the gut–brain axis

A recent study in rats suggested that metformin influences the gut–brain axis [70]. The effect of intraduodenal metformin infusion on duodenal mucosal AMPK production and subsequent hepatic glucose production was assessed. The rats required an increased rate of glucose infusion to maintain euglycaemia during clamp conditions within the first 60 min of intraduodenal metformin administration and had a lower hepatic glucose production. However, when metformin was infused into the hepatic portal vein, no increase in the glucose infusion rate was required, and there was no decrease in hepatic glucose production over the same timeframe [70]. This indicates that duodenal metformin has a direct, pre-absorptive effect on glucose homeostasis in the rat. Based on this finding, it was hypothesised that metformin activates GLP-1 receptors to increase protein kinase A (PKA) activity on intestinal vagal afferents. It was envisaged that the afferents transmit to *N*-methyl-d-aspartate (NMDA) receptors in the nucleus of the solitary tract (NTS), with onward signalling to the efferent fibres of the hepatic vagal nerve, resulting in a reduction in hepatic glucose production. The pre-absorptive effect of metformin was lost when each part of this neuronal pathway was blocked using GLP-1 receptor antagonists or PKA inhibitors, tetracaine, the NMDA receptor blocker MK-801 and hepatic vagotomy. Although these studies were carried out in rat models, they once again highlight the small intestine as a site of action of metformin, and suggest a novel pathway through which metformin may be acting. This mechanism requires further investigation in humans.

## Metformin and bile acids

Metformin increases the bile acid pool within the intestine [65, 71–75], predominantly through reduced ileal absorption [65, 72]. This disruption of the enterohepatic circulation of bile salts has potential consequences for cholesterol homeostasis, entero-endocrine function and glucose homeostasis. It may also contribute to metformin intolerance through alterations in the microbiome and stool consistency. In addition, as discussed previously, the alteration in bile acid absorption may result in increased GLP-1 secretion, in a similar way to that observed with bile acid sequestrants such as colesevelam [76, 77].

Several studies have demonstrated reduced absorption of bile acids in patients receiving metformin treatment and shown this to be a direct effect of metformin on enterocytes [65, 72–75]. Bile acid absorption in the jejunum is a passive, non-saturable and concentration-dependent process, whereas ileal absorption is mainly an active process [73]. FXR is a bile acid sensor involved in ileal absorption of bile acids, as well as the synthesis and secretion of bile acids from the liver. AMPK binds directly to FXR and represses the receptor via direct phosphorylation, resulting in reduced FXR transcriptional activity and, subsequently, reduced bile acid absorption [65]. As an AMPK activator, metformin could potentially exert effects on the bile acid pool via FXR.

A reduction in bile acid absorption has been suggested as a mechanism through which chronic metformin treatment can lower cholesterol [72, 73]. It has also been suggested that an increased luminal bile salt concentration would have an osmotic effect, which could lead to the diarrhoea associated with metformin treatment [72].

## Metformin and the gut microbiome

The gut microbiome and the metagenome (the microbial genome) are areas receiving increasing research attention, and are now considered as environmental factors that contribute to the development of many diseases, including obesity, the metabolic syndrome and type 2 diabetes [78–84]. Large metagenome-wide studies in China and Europe have described gut microbial dysbiosis in association with obesity and type 2 diabetes. Although these two studies differ in terms of the changes they identified, a common finding was a reduction in butyrate-producing bacteria and an increase in opportunistic pathogens [78, 81]. More recently, data from these studies and the Danish MetaHIT project were analysed after controlling for metformin treatment [84]. This identified the reduction in butyrate-producing taxa as a signature of gut microbiome shifts in type 2 diabetes.

**Fig. 2:**
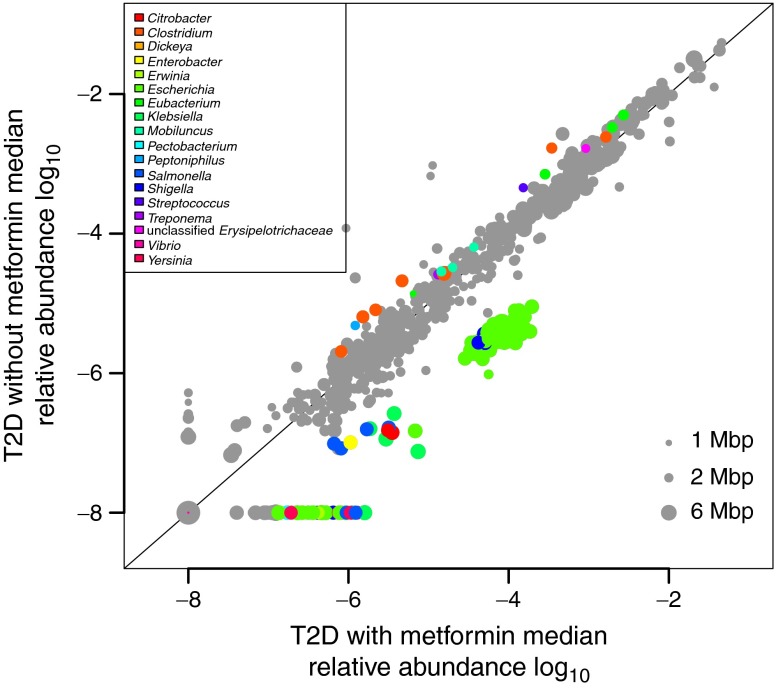
Species abundance in the microbiome of women with type 2 diabetes (T2D) who are treated with metformin (*x*-axis) or who are not metformin treated (*y*-axis). Grey spots represent species that do not differ by metformin exposure. Coloured dots represent species differing by metformin exposure, with the colour representing the bacterial species shown in the key. Figure from Karlsson et al, Gut metagenome in European women with normal, impaired and diabetic glucose control. Nature 2013;498:99–103. Reproduced with permission from Nature Publishing Group

Metformin alters the microbiome in both mice and humans, causing an overall decrease in the bacterial diversity of the mouse microbiome, which contrasts with the effect of a high-fat diet [85, 86]. Studies in humans are more limited, but in a cross-sectional study of the microbiome in women with type 2 diabetes, those treated with metformin had a different microbiome compared with those not treated with the drug (see Fig. 2) [79]. In particular, metformin treatment was accompanied by a marked increase in the bacterium *Akkermansia muciniphila* and an associated increase in mucin-producing goblet cells. In mice, treatment with oligofructose (resulting in an increase in *A. muciniphila*) or direct treatment with *A. muciniphila* has been reported to improve metabolic disorders, possibly by increasing endocannabinoids, which reduce inflammation, modify gut peptide secretion and improve the thickness of the gut mucous barrier [82]. Administration of *A. muciniphila* to mice receiving a high-fat diet improved glucose tolerance [86], suggesting that the effect of metformin on abundance of this species within in the microbiome may indirectly contribute to the glucose-lowering effect of metformin. It has been postulated that an increase in bacteria producing the short-chain fatty acids butyrate and propionate may improve glycaemia [84]. Butyrate and propionate increase intestinal gluconeogenesis. In rodents, increased intestinal gluconeogenesis results in a reduction in hepatic gluconeogenesis, appetite and weight, leading to improved glucose homeostasis.

An alteration in the microbiome by metformin may also be a potential cause of GI intolerance. Burton et al carried out a small open-label crossover study using a GI microbiome modulator (GIMM) or placebo in conjunction with metformin in metformin-intolerant patients with type 2 diabetes [87]. They found that treatment with metformin in combination with GIMM resulted in lower fasting glucose levels, suggesting better tolerance of metformin therapy for longer or at a higher dosage. Therefore, metformin appears to affect the microbiome, and could potentially exert some of its chronic pharmacodynamic effects in this manner, and an individual’s metformin tolerance may be influenced by their microbiome.

## Conclusion

Metformin is a commonly prescribed agent for the treatment of type 2 diabetes. Although it has been in clinical use for decades, its mechanism of action is still under investigation. It has a complex relationship with the gut, both in terms of drug response and drug intolerance. The efficacy of metformin is in part mediated by the gut—potentially by direct effects on glucose uptake and metabolism, by directly or indirectly increasing GLP-1, by an increase in bile acid exposure and by altering the microbiome. These findings, and recent evidence that a delayed-release formulation of metformin with minimal systemic absorption retains its glucose-lowering efficacy [15], suggest that the glucose-lowering effects of metformin are strongly influenced by effects on the gut. The gut is also the site of an important adverse reaction to metformin that often limits metformin dosing or use completely. The mechanisms for this intolerance may relate to altered transport of serotonin or histamine, local metformin concentrations in enterocytes, increased bile acid exposure in the colon or an altered microbiome. Importantly, some of the interindividual variation in metformin tolerance can be explained by common loss-of-function variation in the OCT1 transporter and, potentially, co-prescribed medication. Metformin has been in use for over 60 years. Although a number of studies identifying a key role for metformin in the gut were undertaken many years ago, more recent studies have focused on metformin action in the liver, where it undoubtedly exerts a glucose-lowering effect. Given recent advances in our understanding of metformin transport in the gut, the incretin system, the potential gut–brain axis and the complexities of the microbiome, more research in these areas would doubtless further improve our knowledge and understanding of metformin action and tolerance.

## Abbreviations

AMPK: 5′-AMP-activated protein kinase
CHT: Choline transporter
DPP4: Dipeptidyl peptidase-4
DR: Delayed release
^18^F-FDG: ^18^F-fluorodeoxyglucose
FXR: Farnesoid X receptor
GI: Gastrointestinal
GIMM: Gastrointestinal microbiome modulator
GIP: Gastric inhibitory polypeptide
GLP-1: Glucagon-like peptide 1
GoDARTS: Genetics of Diabetes and Audit Research Tayside Study
5-HT: Serotonin
NMDA: *N*-methyl-d-aspartate
OCT: Organic cation transporter
PET-CT: Positron emission tomography–computed tomography
PKA: Protein kinase A
PMAT: Plasma membrane monoamine transporter
SERT: Serotonin transporter
SGLT1: Sodium–glucose transporter 1
